# Supplementary material for: Mechanisms and Implications of Bacterial Invasion across the Human Skin Barrier
Source: Microbiol Spectr. 2022 May 9;10(3):e02744-21. doi: 10.1128/spectrum.02744-21 (PMC9241919; doi:10.1128/spectrum.02744-21)
Supplement: SUPPLEMENTAL FILE 1 — Supplemental material. Download spectrum.02744-21-s001.pdf, PDF file, 0.4 MB [file spectrum.02744-21-s001.pdf]

Bacterial invasion across the human skin barrier – mechanisms and ensuing tissue degradation.

Supplemental Material

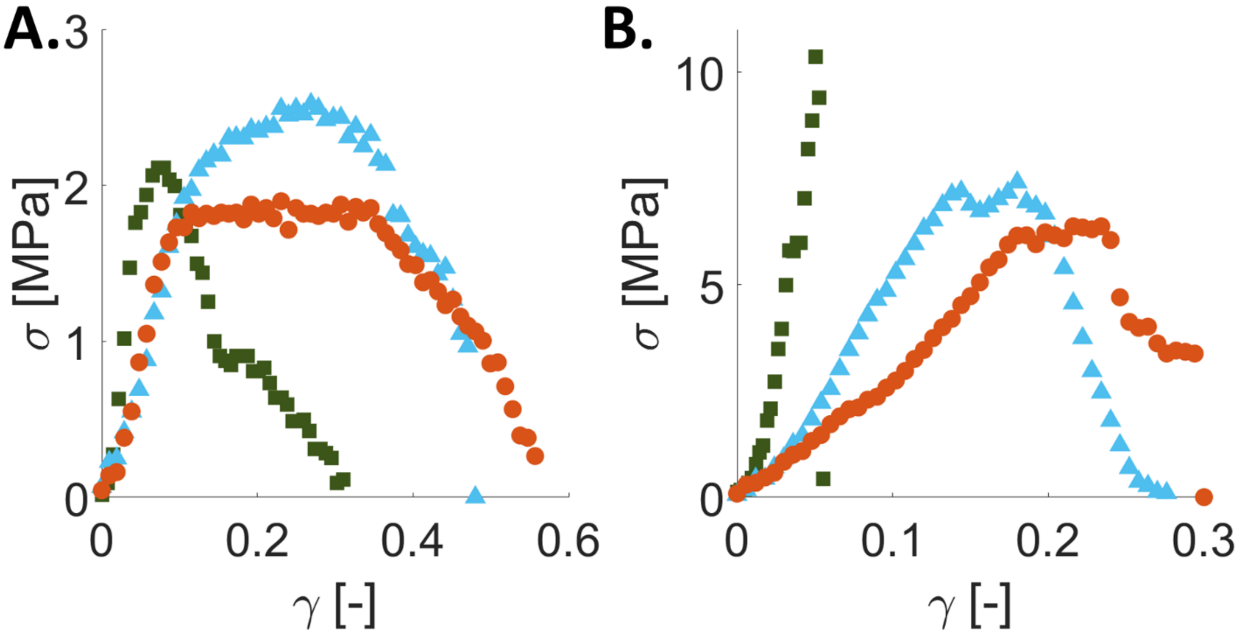

Supplemental Figure 1. *S. aureus* reduces SC plastic deformation at 100% RH. Representative stress-strain plots of stratum corneum samples subjected to: no media immersion (*orange circles*), media immersion (*blue triangles*), and the addition of bacteria (*green squares*) for (A) control and (B) delipid conditions.

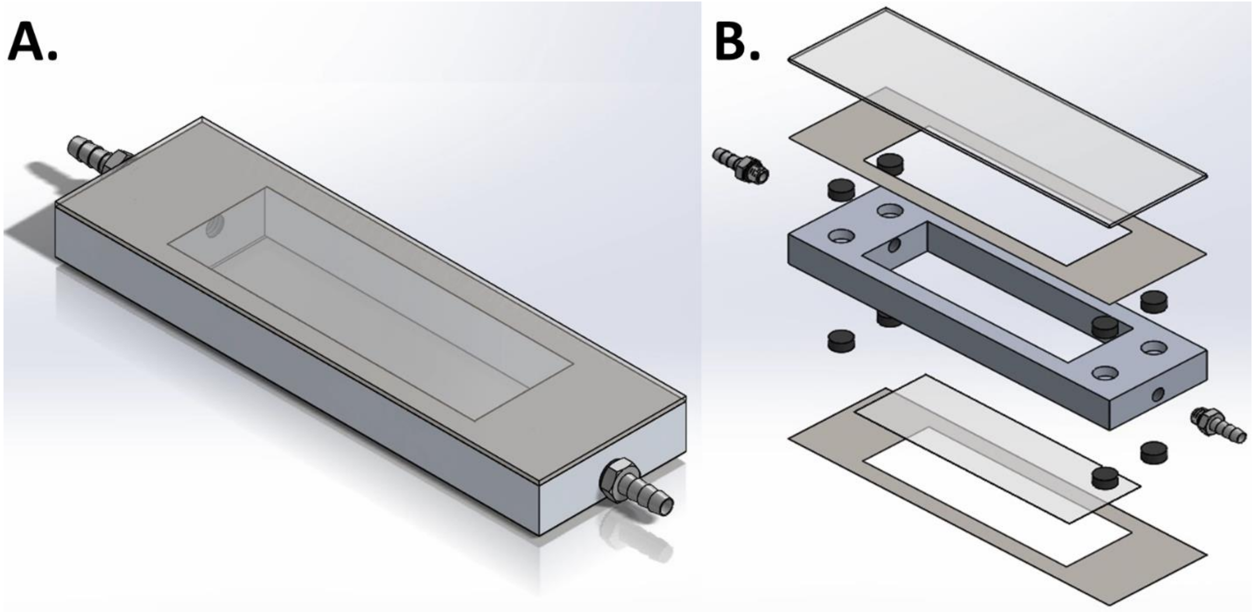

Supplemental Figure 2. Drip chamber schematic. (A) Overview of drip chamber setup (B) Exploded view of drip chamber major components. This device provides a physiologically relevant arrangement that more closely mimics skin temperature and diurnal hydration/dehydration cycles to study bacteria-skin interactions.

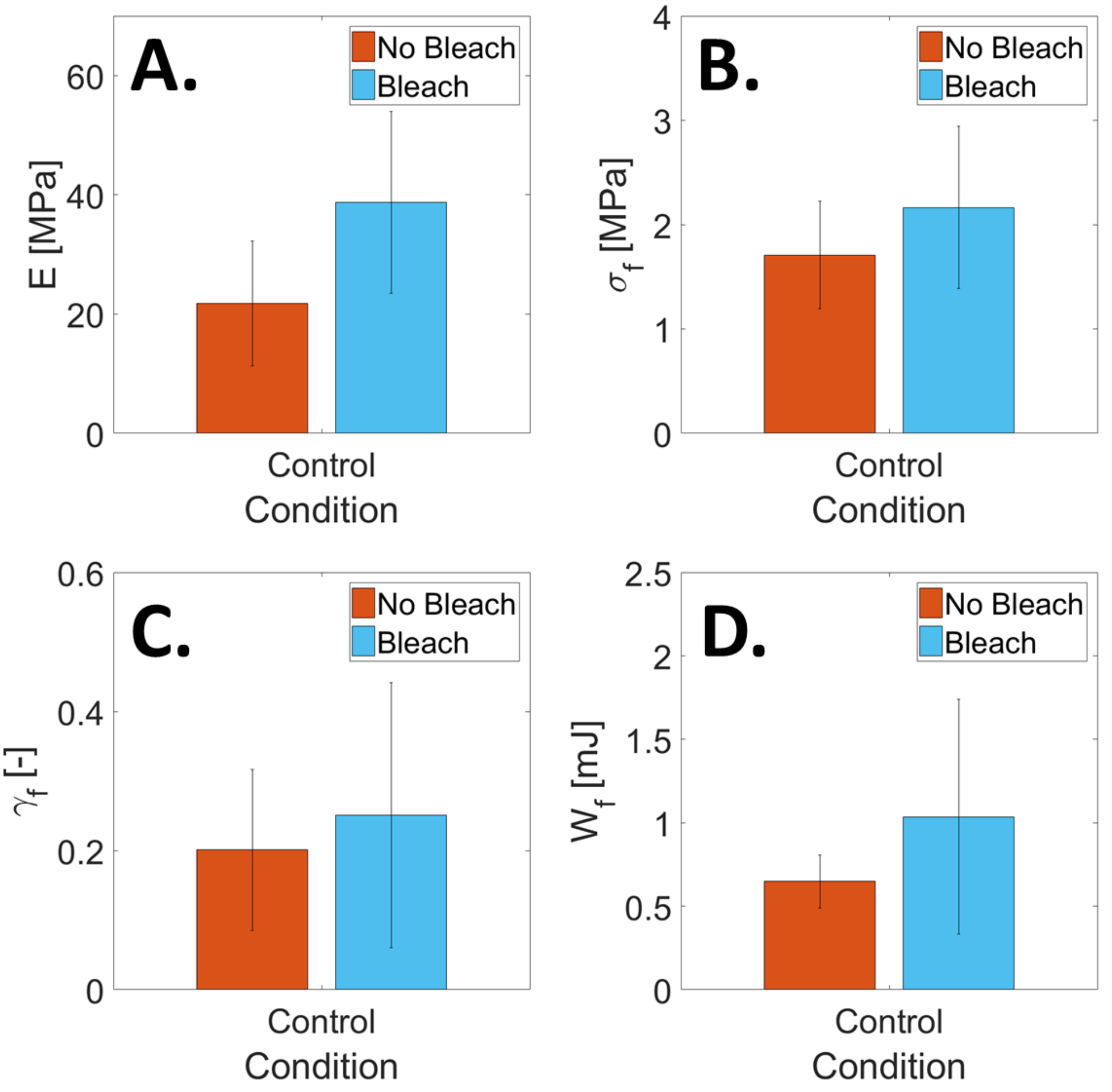

Supplemental Figure 3. Bleach at 2.5 ml/L does not affect the mechanical properties of SC. Average (A) elastic modulus, E, (B) fracture stress, σ_f, (C) fracture strain, γ_f, and (D) work of fracture, W_f for control samples (*orange bars*) and samples incubated in bleach for 30 mins (*blue bars*). Bars denote average values of $4 \leq n \leq 9$ individual sample measurements for each exposure condition. Error bars denote standard deviations.
